# Emotional Intelligence and Nurses’ Job Performance: Evidence From a Ghanaian Teaching Hospital

**DOI:** 10.1155/jonm/2359356

**Published:** 2026-09-14

**Authors:** Emmanuel Duncan

**Affiliations:** ^1^ Department of Human Resource Management, University of Cape Coast, Cape Coast, Ghana, ucc.edu.gh

**Keywords:** emotional competence, emotional intelligence, health care, interpersonal relationship, job performance, nursing, psychology, social skills

## Abstract

Emotional intelligence may be relevant to nurses’ communication, emotional regulation, teamwork and job performance. Evidence on individual emotional intelligence dimensions among nurses in Ghana remains limited. This study examined the associations between self‐awareness, self‐management, social awareness, relationship management and nurses’ job performance at Cape Coast Teaching Hospital, Ghana. A cross‐sectional analytical study was conducted among nurses at Cape Coast Teaching Hospital. A simple random sample of 267 eligible nurses was selected, of whom 219 completed the questionnaire (82.0% response rate). Emotional intelligence was measured using an adapted instrument based on Goleman’s Emotional Competency Framework, while job performance was assessed using the Employee Job Performance Scale. Exploratory factor analysis was conducted using principal axis factoring. Factorability was assessed using the Kaiser–Meyer–Olkin measure and Bartlett’s test of sphericity, and internal consistency was assessed using Cronbach’s alpha. Pearson correlation and multiple linear regression analyses were conducted. All four emotional intelligence dimensions were positively correlated with job performance. In Model 1, self‐awareness (*β* = 0.289, *p* < 0.001) and relationship management (*β* = 0.265, *p* = 0.001) were positively associated with job performance, while self‐management (*β* = 0.099, *p* = 0.213) and social awareness (*β* = −0.026, *p* = 0.770) were not independently associated. The model explained 26.5% of the variance in job performance (*R* ^2^ = 0.265; adjusted *R* ^2^ = 0.252). After adjustment for gender, nursing education, work experience and nursing category, self‐awareness (*β* = 0.258, *p* = 0.001) and relationship management (*β* = 0.260, *p* = 0.001) remained significant. The adjusted model explained 31.4% of the variance (*R* ^2^ = 0.314; adjusted *R* ^2^ = 0.271). Self‐awareness and relationship management were independently associated with nurses’ self‐reported job performance. Longitudinal and intervention studies are needed to determine whether changes in these competencies are associated with subsequent changes in nursing performance and patient‐care outcomes.

## 1. Introduction

Emotional intelligence refers to the ability to recognise, understand, regulate and appropriately use one’s own emotions and those of others. In nursing practice, these competencies are relevant because nurses work in emotionally demanding situations that require sound judgement, empathy, communication, emotional regulation and effective interpersonal interaction. These capabilities are associated with effective patient care, teamwork, communication and professional practice [1–3]. Emotional intelligence has also been associated with patient‐centred care, effective communication, teamwork and clinical decision‐making among nurses [4].

The relevance of emotional intelligence is particularly evident in Ghanaian public hospitals, where nurses work within demanding clinical environments characterised by increasing patient demand, heavy workloads, staff shortages, and substantial emotional demands [5]. Cape Coast Teaching Hospital, the principal referral hospital for the Central Region of Ghana, serves a large patient population and operates within a demanding nurse‐to‐patient environment [6]. In such settings, nurses must manage their own emotional responses while communicating with patients and relatives, collaborating with colleagues, and maintaining professional standards during demanding clinical encounters. Previous studies in Ghanaian healthcare facilities have also identified concerns relating to nurse‐patient interactions, communication, and professional attitudes, highlighting the relevance of emotional competencies to healthcare quality [7–9].

Existing evidence has linked emotional intelligence with job performance, interpersonal relationships and adaptability in demanding work environments [10–12]. Within nursing, studies have reported positive associations between emotional intelligence and job performance, although findings have not been consistent across settings [13–15]. These differences suggest that the relationship between emotional intelligence and job performance may vary across professional and healthcare contexts.

Despite the growing international literature, empirical evidence on emotional intelligence among nurses in Ghana remains limited. In addition, existing research has often examined emotional intelligence as a composite construct, providing less evidence on how individual emotional intelligence dimensions are associated with nurses’ job performance [12]. This leaves limited evidence on whether specific emotional competencies have different associations with nursing performance in the Ghanaian hospital context. Examining the dimensions separately is, therefore, relevant to nursing management because it can indicate which competencies are more closely associated with nurses’ performance and may warrant attention in professional development.

Goleman’s Mixed Competency Model provides a relevant framework for examining these dimensions. The model conceptualises emotional intelligence as four interrelated competencies: self‐awareness, self‐management, social awareness, and relationship management [16]. These competencies correspond closely with the interpersonal, emotional, and behavioural demands of nursing practice. Self‐awareness concerns the recognition of one’s emotions, strengths and limitations; self‐management concerns the regulation of emotions and responses under pressure; social awareness concerns understanding the emotions and needs of others; and relationship management concerns building productive relationships, managing conflict and promoting effective teamwork. In a teaching hospital, these competencies are relevant to communication with patients and colleagues, collaboration within multidisciplinary teams, professional conduct and coordinated patient care.

Accordingly, this study examined the associations between self‐awareness, self‐management, social awareness, relationship management, and nurses’ job performance at Cape Coast Teaching Hospital, Ghana. Drawing on Goleman’s Mixed Competency Model and the existing empirical evidence, the study hypothesised that each emotional intelligence dimension would be positively associated with nurses’ job performance.

### 1.1. Theoretical Framework and Hypothesis Development

This study is grounded in Goleman’s Mixed Competency Model, which conceptualises emotional intelligence as a set of personal and social competencies associated with workplace effectiveness [16]. The model comprises four interrelated dimensions: self‐awareness, self‐management, social awareness and relationship management. The model is relevant to nursing because nurses work in emotionally demanding clinical environments where professional performance involves managing personal emotions, responding appropriately to patients’ needs, communicating with colleagues, and coordinating care within multidisciplinary teams. At Cape Coast Teaching Hospital, these competencies are relevant to the interpersonal and behavioural demands associated with nursing practice and job performance. The theoretical framework, therefore, provides a basis for examining the four emotional intelligence dimensions separately rather than treating emotional intelligence as a single composite construct.

### 1.2. Self‐Awareness and Nurses’ Job Performance

Self‐awareness refers to the ability to recognise and understand one’s emotions, strengths, limitations, and the ways in which emotional states may shape behaviour [16]. In nursing practice, self‐awareness provides a basis for recognising emotional reactions during demanding clinical encounters and considering how these reactions may affect communication, professional conduct and decision‐making. Nurses who recognise their emotional responses may be better positioned to maintain appropriate professional behaviour, respond constructively to feedback and remain attentive to patient and workplace demands. These capabilities provide a theoretical basis for expecting an association between self‐awareness and job performance.

Previous studies have reported positive associations between self‐awareness and job performance among healthcare workers and other service professionals, although findings have not been consistent across settings [3, 17–20]. Based on Goleman’s model and the demands of nursing practice, the following hypothesis was formulated:

### 1.3. H_1_: Self‐Awareness is Positively Associated With Nurses’ Job Performance at Cape Coast Teaching Hospital.

#### 1.3.1. Self‐Management and Nurses’ Job Performance

Self‐management refers to the ability to regulate emotional responses, exercise self‐control, adapt to changing circumstances, and maintain an appropriate behavioural response in challenging situations [16, 21]. Nursing practice frequently involves changing clinical conditions, demanding workloads, emotionally difficult patient encounters and interactions with patients, relatives and other healthcare professionals. The capacity to manage emotional responses may, therefore, support nurses’ ability to maintain professional standards and respond appropriately under pressure.

From the perspective of Goleman’s model, self‐management represents the regulation component of personal emotional competence. Its relevance to nursing performance lies in the expectation that effective regulation of emotions may support consistent professional behaviour, communication and decision‐making during demanding clinical situations. Previous studies have reported mixed findings regarding the association between self‐management and job performance. Some have reported positive associations [3, 22], whereas others have found no statistically significant relationship [20, 23]. Based on the theoretical relevance of emotional regulation to nursing practice, the following hypothesis was proposed:

### 1.4. H_2_: Self‐Management is Positively Associated With Nurses’ Job Performance at Cape Coast Teaching Hospital.

#### 1.4.1. Social Awareness and Nurses’ Job Performance

Social awareness refers to the ability to recognise and understand the emotions, needs and concerns of other people, including empathy and awareness of the social environment [16, 24]. This competency has particular relevance to nursing because patient care requires nurses to recognise patients’ emotional and interpersonal needs, communicate appropriately and respond to concerns within the clinical environment.

Social awareness may also support effective interactions with colleagues by helping nurses recognise the perspectives and emotional states of other members of the healthcare team. In a teaching hospital, where patient care involves interaction among nurses, physicians, allied health professionals, patients and relatives, the ability to understand others’ needs may be relevant to communication, teamwork and coordination of care. These considerations provide a theoretical basis for expecting social awareness to be associated with nurses’ job performance.

Empirical findings have been mixed. Some studies have reported positive associations between social awareness and job performance [18, 25, 26], whereas others have reported nonsignificant relationships [19, 27]. Accordingly, the following hypothesis was formulated:

### 1.5. H_3_: Social Awareness is Positively Associated With Nurses’ Job Performance at Cape Coast Teaching Hospital

#### 1.5.1. Relationship Management and Nurses’ Job Performance

Relationship management refers to the ability to build productive relationships, manage interpersonal conflict, communicate effectively, influence others appropriately and promote teamwork [16, 28]. Relationship management is particularly relevant to nursing because nurses work continuously with patients, relatives, physicians and other healthcare professionals. Effective interpersonal relationships can support communication, collaboration, conflict management and coordination of patient care.

Within Goleman’s model, relationship management represents the application of emotional and social competencies to interactions with others. In nursing practice, this competency may, therefore, be associated with job performance through effective teamwork, communication and coordination of responsibilities. The collaborative nature of care in a teaching hospital provides a context in which interpersonal competencies are particularly relevant to nurses’ day‐to‐day performance.

Previous studies have reported positive associations between relationship management and employee performance in healthcare and other organisational settings [17, 19, 22, 29]. Based on the theoretical framework and empirical evidence, the following hypothesis was proposed:

### 1.6. H_4_: Relationship Management is Positively Associated With Nurses’ Job Performance at Cape Coast Teaching Hospital.

#### 1.6.1. Conceptual Framework

**FIGURE 1:**
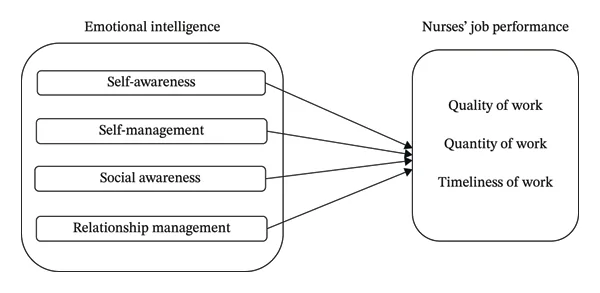
Conceptual framework. Source: Author’s construct (2021).

The conceptual framework positions self‐awareness, self‐management, social awareness, and relationship management as the four emotional intelligence dimensions examined in relation to nurses’ job performance. The framework reflects the theoretical proposition that each dimension represents a distinct but interrelated emotional competency that may be associated with nurses’ performance in the clinical environment. Consistent with Na‐Nan et al. [30] and Cox‐Kelley et al. [31], nurses’ job performance was assessed using indicators of work quality, work quantity and work timeliness. Figure 1 presents the conceptual framework guiding the study.

### 1.7. Conceptual Framework

Figure 1 illustrates the hypothesised associations between emotional intelligence dimensions (self‐awareness, self‐management, social awareness and relationship management) and nurses’ job performance (quality, quantity and timeliness of work) at Cape Coast Teaching Hospital, Ghana.

## 2. Materials and Methods

### 2.1. Design and Setting

A cross‐sectional analytical design was adopted to examine the associations between emotional intelligence dimensions and nurses’ job performance at Cape Coast Teaching Hospital, the principal referral hospital for Ghana’s Central Region. The hospital serves a large and diverse patient population and experiences a high nurse workload, with an average nurse‐to‐patient admission ratio of approximately 1:20, making it an appropriate setting for examining the relationship between emotional intelligence and job performance in a demanding clinical environment [6].

### 2.2. Participants and Sampling

The target population comprised 799 registered nurses, including professional nurses, midwives, and enrolled nurses employed at Cape Coast Teaching Hospital [6]. The required sample size of 267 participants was determined using Yamane’s [32] sample size formula. Eligible nurses were identified based on the study inclusion criteria and constituted the sampling frame. Each eligible nurse was assigned a unique identification number, and the required participants were selected using simple random sampling. This procedure provided each eligible nurse within the sampling frame with an opportunity to be selected.

Eligible participants were registered nurses, professional nurses, enrolled nurses and midwives who were permanently employed at the hospital, had worked for at least 6 months, were directly involved in patient care, and were available during the data collection period. Nurses on annual leave, study leave, maternity leave or extended sick leave, temporary staff, student nurses and administrative nursing personnel without direct clinical responsibilities were excluded. Recruitment covered the hospital’s major inpatient and outpatient clinical units, including medical, surgical, paediatric, maternity, emergency and specialist departments. A total of 267 questionnaires were distributed, of which 219 were completed and included in the analysis, representing a response rate of 82.0%.

The remaining 48 questionnaires were not returned during the data collection period, primarily because some eligible nurses were on leave or off duty. The study did not record separate refusal rates. Demographic information was not collected for nonrespondents; therefore, comparisons between respondents and nonrespondents could not be undertaken. Ward‐ and shift‐specific response rates were also not separately recorded.

### 2.3. Instruments

Two structured questionnaires were used for data collection. The first measured emotional intelligence, while the second measured nurses’ job performance.

Emotional intelligence: Emotional intelligence was measured using an adapted version of the Emotional Intelligence Self‐Assessment Questionnaire developed by Mohapel [33], based on Goleman’s Emotional Competency Framework [16]. The original instrument comprised 27 items measuring four dimensions of emotional intelligence: self‐awareness, self‐management, social awareness and relationship management. The instrument was adapted to improve its relevance to the Ghanaian nursing context while preserving the original constructs.

The emotional intelligence items were rated on a 5‐point Likert scale ranging from 1 (*Never*) to 5 (*Always*), with higher scores indicating higher levels of the respective emotional intelligence dimension. Composite mean scores were calculated for each dimension and used in the subsequent statistical analyses.

Nurses’ job performance. Nurses’ job performance was assessed using the 13‐item Employee Job Performance Scale developed by Na‐Nan et al. [30]. The scale measures three aspects of job performance: work quantity, work quality and work timeliness. Items were rated on a 5‐point Likert scale ranging from 1 (*Strongly disagree*) to 5 (*Strongly agree*), with higher scores indicating higher levels of job performance.

### 2.4. Instrument Adaptation and Validity Assessment

The instruments were adapted for use in the Ghanaian nursing context while retaining the constructs represented in the original measures. The adapted questionnaires were reviewed by the study supervisor before administration and were subsequently vetted and approved by the Ethical Review Board of Cape Coast Teaching Hospital.

No separate pilot study was conducted before the main data collection. Instead, construct validity was assessed using factor analysis based on the study data. The factorability of the constructs was examined using the Kaiser–Meyer–Olkin (KMO) measure and Bartlett’s test of sphericity. Item retention was assessed using factor loadings, with a loading of 0.50 used as the acceptable threshold. Items that did not meet the specified criterion were excluded from the final measurement structure.

The final measurement structure comprised five items for self‐awareness, six for self‐management, six for social awareness, five for relationship management and eleven for nurses’ job performance. Internal consistency was assessed using Cronbach’s alpha. The factorability, item‐retention and reliability results are presented in Table 1, while the factor loadings of the retained items are presented in Table 2.

**TABLE 1:** Exploratory factor analysis and reliability.

Construct	Retained items	KMO	Bartlett’s *χ* ^2^	Df	*p* Values
Self‐awareness	5	0.707	269.366	21	< 0.001
Self‐management	6	0.820	273.228	15	< 0.001
Social awareness	6	0.848	414.176	21	< 0.001
Relationship management	5	0.787	417.387	21	< 0.001
Nurses’ job performance	11	0.861	1120.771	78	< 0.001

*Note:* Source: Authors’ fieldwork data (2021).

**TABLE 2:** Factor loadings of the retained items.

Construct	Item	Factor loading
Self‐awareness	SA1	0.706
	SA2	0.625
	SA4	0.561
	SA6	0.632
	SA7	0.593

Self‐management	SM8	0.603
	SM9	0.527
	SM10	0.592
	SM11	0.704
	SM12	0.572
	SM13	0.578

Social awareness	SEA14	0.632
	SEA15	0.684
	SEA16	0.679
	SEA18	0.538
	SEA19	0.668
	SEA20	0.649

Relationship management	RM21	0.582
	RM22	0.749
	RM23	0.602
	RM24	0.677
	RM27	0.580

Nurses’ job performance	NP2	0.582
	NP3	0.536
	NP4	0.515
	NP5	0.646
	NP6	0.590
	NP8	0.652
	NP9	0.620
	NP10	0.697
	NP11	0.729
	NP12	0.700
	NP13	0.660

*Note:* Source: Authors’ fieldwork data (2021).

### 2.5. Data Collection Procedure

Data collection was conducted over a 2‐week period across morning, afternoon, and evening shifts to accommodate the work schedules of nurses across the participating clinical units. The researcher distributed the questionnaires to the selected nurses and explained the purpose of the study and the instructions for completing the questionnaires. Because data collection took place in a busy clinical environment, some participants completed the questionnaires immediately, while others requested that the researcher return later when their clinical duties permitted.

To protect the confidentiality of responses during questionnaire collection, participants placed completed questionnaires in envelopes before handing them to the person in charge or shift leader of their respective units. The researcher subsequently collected the completed questionnaires from the designated unit personnel. This procedure allowed participants to submit their completed questionnaires without exposing their responses to unit personnel.

Data collection was conducted during different shifts over the 2‐week period to accommodate nurses who were unavailable during the researcher’s initial visits.

### 2.6. Ethical Considerations

Ethical approval was obtained from the Institutional Review Board of Cape Coast Teaching Hospital (Approval No. CCTHERC/EC/2021/084) before data collection commenced. Participation was voluntary, and written informed consent was obtained from all respondents. Participants were assured that their responses would remain anonymous and confidential, no personally identifying information was collected, completed questionnaires were used solely for research purposes and respondents were informed of their right to withdraw from the study at any stage without penalty.

## 3. Results

A total of 219 completed questionnaires were analysed, representing an 82.0% response rate. As shown in Table 3, most respondents were female (71.2%) and single (58.9%). Regarding educational attainment, 47.5% held diploma qualifications, 26.5% had qualifications from a nursing training college, 22.4% held bachelor’s degrees and 3.7% held master’s degrees. General nurses constituted the largest occupational group (56.6%), followed by midwives (27.9%) and professional nurses (15.5%). In terms of work experience, 53.4% had between 1 and 5 years of professional experience, while 28.3% had less than 1 year, 11.0% had 6–10 years and 7.3% had 11 years or more. Most respondents worked the morning shift (64.4%), followed by the afternoon (21.9%) and night (13.7%) shifts.

**TABLE 3:** Demographic characteristics of respondents.

Variable	Category	Frequency (*N*)	Percentage (%)
Gender	Female	156	71.2
	Male	63	28.8

Marital status	Single	129	58.9
	Married	85	38.8
	Divorced/Widowed	5	2.3

Nursing education	Diploma	104	47.5
	Nursing Training College	58	26.5
	Bachelor’s degree	49	22.4
	Master’s Degree	8	3.7

Work experience	Less than 1 year	62	28.3
	1–5 years	117	53.4
	6–10 years	24	11.0
	11 years and above	16	7.3

Nursing category	General Nurses	124	56.6
	Midwives	61	27.9
	Professional Nurses	34	15.5

Shift duty	Morning	141	64.4
	Afternoon	48	21.9
	Night	30	13.7

*Note:* Source: Authors’ fieldwork data (2021).

### 3.1. Exploratory Factor Analysis and Reliability

Exploratory factor analyses were conducted separately for the five study constructs using principal axis factoring. The results indicated that the data were suitable for factor analysis across all five constructs. The KMO values ranged from 0.707 for self‐awareness to 0.861 for nurses’ job performance, while Bartlett’s tests of sphericity were statistically significant across all constructs (*p* < 0.001). The analysis resulted in the retention of five self‐awareness items, six self‐management items, six social‐awareness items, five relationship‐management items and eleven nurses’ job performance items. The detailed factorability and reliability results are presented in Table 1.

### 3.2. Factor Loadings and Variance

The factor loadings for the retained items are presented in Table 2. The extracted factors accounted for 27.5% of the variance in self‐awareness, 35.8% in self‐management, 38.6% in social awareness, 35.3% in relationship management and 36.8% in nurses’ job performance. The retained items which showed factor loadings above the threshold of 0.5 were considered acceptable factor loadings [34].

Table 4 presents the descriptive statistics and internal consistency of the study variables. The mean scores for the four emotional intelligence dimensions ranged from 3.822 for self‐management to 3.896 for self‐awareness. Nurses’ job performance had a mean score of 3.865 (SD = 0.531). The measures demonstrated acceptable internal consistency, with Cronbach’s alpha coefficients ranging from 0.738 for self‐awareness to 0.878 for nurses’ job performance.

**TABLE 4:** Descriptive statistics and reliability.

Variable	M	SD	Cronbach’s *α*
Self‐awareness	3.896	0.618	0.738
Self‐management	3.822	0.643	0.766
Social awareness	3.887	0.607	0.805
Relationship management	3.892	0.603	0.769
Nurses’ job performance	3.865	0.531	0.878

*Note:* Source: Authors’ fieldwork data (2021).

Table 5 shows that all four emotional intelligence dimensions were positively associated with nurses’ job performance. Self‐awareness had the strongest bivariate association with job performance (*r* = 0.437, *p* < 0.001), followed by relationship management (*r* = 0.422, *p* < 0.001), self‐management (*r* = 0.373, *p* < 0.001) and social awareness (*r* = 0.368, *p* < 0.001). The emotional intelligence dimensions were also positively correlated with one another, with coefficients ranging from 0.421 to 0.645. These correlations indicate associations among the constructs but do not establish causal relationships.

**TABLE 5:** Correlations of the study variables.

Variable	1	2	3	4	5
1. Self‐awareness	1				
2. Self‐management	0.518^∗∗∗^	1			
3. Social awareness	0.559^∗∗∗^	0.624^∗∗∗^	1		
4. Relationship management	0.421^∗∗∗^	0.532^∗∗∗^	0.645^∗∗∗^	1	
5. Nurses’ job performance	0.437^∗∗∗^	0.373^∗∗∗^	0.368^∗∗∗^	0.422^∗∗∗^	1

*Note:* Source: Authors’ fieldwork data (2021).

^∗∗∗^ *p* < 0.001.

### 3.3. Multiple Regression Analysis

A multiple linear regression analysis was conducted to examine the associations between the four emotional intelligence dimensions and nurses’ job performance. Model 1 included self‐awareness, self‐management, social awareness and relationship management as the predictor variables. Model 2 extended Model 1 by adding the available demographic and work‐related covariates, namely, gender, nursing education, work experience and nursing category.

Table 6 presents the results of the two regression models. Model 1, which included the four emotional intelligence dimensions, was statistically significant, *F*(4, 214) = 19.32, *p* < 0.001, and explained 26.5% of the variance in nurses’ job performance (*R* ^2^ = 0.265; adjusted *R* ^2^ = 0.252). Self‐awareness was positively associated with job performance (*β* = 0.289, *p* < 0.001), as was relationship management (*β* = 0.265, *p* = 0.001). Self‐management (*β* = 0.099, *p* = 0.213) and social awareness (*β* = −0.026, *p* = 0.770) were not independently associated with job performance in Model 1.

**TABLE 6:** Multiple regression of emotional intelligence dimensions on nurses’ job performance.

Predictor	Model 1 B	Model 1 *β*	Model 1 *p*	Model 2 B	Model 2 *β*	Model 2 *p*
Self‐awareness	0.248	0.289	< 0.001	0.222	0.258	0.001
Self‐management	0.089	0.099	0.213	0.087	0.096	0.226
Social awareness	−0.024	−0.026	0.770	0.013	0.014	0.874
Relationship management	0.248	0.265	< 0.001	0.243	0.260	0.001

*Note:* Model 1: *R* ^2^ = 0.265, adjusted *R* ^2^ = 0.252, *F*(4, 214) = 19.32, *p* < 0.001. Model 2: *R* ^2^ = 0.314, adjusted *R* ^2^ = 0.271, *F*(13, 205) = 7.23, *p* < 0.001. Source: Authors’ fieldwork data (2021).

Model 2 incorporated gender, nursing education, work experience and nursing category as covariates. The adjusted model was statistically significant, *F*(13, 205) = 7.23, *p* < 0.001, and explained 31.4% of the variance in nurses’ job performance (*R* ^2^ = 0.314; adjusted *R* ^2^ = 0.271). After adjustment, self‐awareness remained positively associated with job performance (*β* = .258, *p* = 0.001), as did relationship management (*β* = 0.260, *p* = 0.001). Self‐management (*β* = 0.096, *p* = 0.226) and social awareness (*β* = 0.014, *p* = .874) remained nonsignificant.

Regression diagnostics indicated no evidence of problematic multicollinearity, heteroscedasticity, nonlinearity or highly influential observations. The Durbin–Watson statistic was 1.58, providing no indication of a substantial departure from residual independence.

## 4. Discussion

### 4.1. Self‐Awareness and Nurses’ Job Performance

Self‐awareness showed a positive and statistically significant association with nurses’ job performance in both the bivariate correlation and multivariable regression analyses. In the regression model containing the four emotional intelligence dimensions, self‐awareness remained independently associated with job performance (*β* = 0.289, *p* < 0.001, 95% CI [0.124, 0.373]). The association also remained statistically significant after adjustment for gender, nursing education, work experience and nursing category (*β* = 0.258, *p* = 0.001, 95% CI [0.095, 0.348]). This consistency across the bivariate and adjusted analyses suggests that self‐awareness had a distinct association with nurses’ self‐reported job performance in the study sample.

This finding is consistent with Goleman’s Mixed Competency Model, which positions self‐awareness as a foundational emotional competency involving recognition of one’s emotions, strengths, limitations and their relationship with behaviour [16]. In nursing practice, awareness of one’s emotional state may help nurses recognise how emotional responses arise during demanding patient encounters and workplace interactions. Such awareness may support reflective practice, professional communication, and appropriate responses to challenging situations. These mechanisms provide a plausible explanation for the observed association, although the cross‐sectional design does not establish that self‐awareness causes higher job performance.

The finding is consistent with previous studies that reported positive associations between self‐awareness and job performance among healthcare workers and employees in other service settings [3, 17, 18, 22]. However, it differs from studies by Oguns and Uhunamure [19] and Shahzad et al. [20], which reported nonsignificant relationships. These differences may reflect variation in professional settings, measurement approaches, organisational conditions and the characteristics of the populations studied.

The finding has particular relevance to the nursing context at Cape Coast Teaching Hospital, where nurses work in demanding clinical conditions involving high patient workloads and emotionally challenging interactions. The ability to recognise one’s emotional responses may be relevant to maintaining professional conduct and effective communication under such conditions. However, self‐awareness represents only one component of job performance, and Model 1 explained 26.5% of the variance in nurses’ job performance (*R* ^2^ = 0.265; adjusted *R* ^2^ = 0.252). After inclusion of the available demographic and work‐related covariates, Model 2 explained 31.4% of the variance (*R* ^2^ = 0.314; adjusted *R* ^2^ = 0.271).

### 4.2. Self‐Management and Nurses’ Job Performance

Self‐management was positively associated with nurses’ job performance at the bivariate level, but it was not independently associated with job performance in the multivariable regression model. In the correlation analysis, self‐management showed a significant positive relationship with job performance (*r* = 0.373, *p* < 0.001). However, after the four emotional intelligence dimensions were entered simultaneously, the association was no longer statistically significant (*β* = 0.099, *p* = 0.213, 95% CI [−0.052, 0.230]). The association also remained nonsignificant in the adjusted model that included gender, nursing education, work experience and nursing category (*β* = 0.096, *p* = 0.226, 95% CI [−0.054, 0.228]).

This finding suggests that the positive bivariate relationship between self‐management and job performance did not represent a distinct association after the overlap among the emotional intelligence dimensions was considered. The four dimensions were positively correlated with one another, indicating that nurses who reported stronger self‐management also tended to report higher levels of other emotional competencies. The attenuation of the self‐management association in the multivariable model may, therefore, reflect shared variance among the emotional intelligence dimensions.

The finding is consistent with Shahzad et al. [20] and Shahzad and Alvi [23], who reported nonsignificant relationships between self‐management and employee performance. However, it differs from Gontur and Dekom [22] and Vestal [3], who reported significant positive associations. These differences may reflect variations in occupational settings, measurement approaches, organisational conditions and the way other emotional intelligence competencies are accounted for in the analysis.

Within nursing practice, self‐management remains relevant to emotional regulation, adaptability and maintaining professional behaviour during challenging situations. However, the present findings do not provide evidence that self‐management has an independent statistical association with nurses’ self‐reported job performance after accounting for the other emotional intelligence dimensions. This distinction is important because the significant bivariate relationship should not be interpreted as evidence of an independent association.

### 4.3. Social Awareness and Nurses’ Job Performance

Social awareness was positively associated with nurses’ job performance at the bivariate level, but it was not independently associated with job performance in the multivariable analysis. The correlation analysis showed a significant positive relationship between social awareness and job performance (*r* = 0.368, *p* < 0.001). However, after the four emotional intelligence dimensions were entered simultaneously into the regression model, social awareness was not statistically significant (*β* = −0.026, *p* = 0.770, 95% CI [−0.182, 0.135]). The association also remained nonsignificant after adjustment for gender, nursing education, work experience and nursing category (*β* = 0.014, *p* = 0.874, 95% CI [−0.151, 0.177]).

The difference between the bivariate and multivariable findings may reflect the substantial overlap among the emotional intelligence dimensions. Social awareness was positively correlated with self‐awareness (*r* = 0.559), self‐management (*r* = 0.624) and relationship management (*r* = 0.645). Thus, the significant bivariate association between social awareness and job performance may partly reflect variance shared with other emotional intelligence competencies. When the dimensions were considered simultaneously, social awareness did not account for a statistically significant unique association with job performance.

The finding contrasts with studies by Bahraminan et al. [25], Ifelebuegu et al. [26], and Mohamad and Jais [18], which reported positive associations between social awareness and performance, but is consistent with findings reported by Agarwal [27] and Oguns and Uhunamure [19]. The differences across studies may be related to differences in professional settings, study populations, measurement approaches and whether other emotional intelligence dimensions were considered simultaneously.

Social awareness remains relevant to nursing practice because understanding patients’ emotions, needs and concerns can support empathetic communication and patient‐centred interactions. However, the present findings do not demonstrate an independent statistical association between social awareness and nurses’ self‐reported job performance after accounting for the other emotional intelligence dimensions. This finding should, therefore, be interpreted as an association within the study sample rather than evidence that social awareness causes changes in job performance.

### 4.4. Relationship Management and Nurses’ Job Performance

Relationship management was positively associated with nurses’ job performance in both the bivariate and multivariable analyses. The bivariate analysis showed a significant positive correlation between relationship management and job performance (*r* = 0.422, *p* < 0.001). In the multivariable regression model, relationship management remained independently associated with job performance (*β* = 0.265, *p* = 0.001, 95% CI [0.103, 0.394]). The association remained statistically significant after adjustment for gender, nursing education, work experience and nursing category (*β* = 0.260, *p* = 0.001, 95% CI [0.097, 0.390]).

This finding is consistent with Goleman’s Mixed Competency Model, which describes relationship management in terms of developing productive relationships, managing conflict, influencing others and promoting effective interactions [16]. These competencies have direct relevance to nursing practice, where effective communication and collaboration are required in interactions with patients, relatives, colleagues and other healthcare professionals. The present finding is also consistent with previous studies that reported positive associations between relationship management and employee performance in healthcare and other organisational settings [17, 19, 22, 29].

The persistence of the association after accounting for the other emotional intelligence dimensions suggests that relationship management had a distinct association with nurses’ self‐reported job performance in this sample. This may be particularly relevant in a teaching hospital, where nursing work involves continuous coordination across professional groups and frequent interpersonal interactions. However, the cross‐sectional design does not permit a causal interpretation of this association. The finding, therefore, supports further investigation of relationship management in longitudinal and intervention‐based nursing research.

## 5. Conclusion

This study examined the associations between four dimensions of emotional intelligence and nurses’ self‐reported job performance at Cape Coast Teaching Hospital, Ghana. All four emotional intelligence dimensions were positively associated with job performance at the bivariate level. However, in the multivariable analysis, only self‐awareness and relationship management remained statistically significant, while self‐management and social awareness were not independently associated with job performance. These findings indicate that the emotional intelligence dimensions may have different levels of association with nurses’ job performance when considered simultaneously.

The findings provide empirical evidence on the individual dimensions of emotional intelligence among nurses in a Ghanaian teaching hospital and highlight self‐awareness and relationship management as competencies warranting further attention in nursing management and professional development. However, the cross‐sectional design does not permit causal conclusions about whether strengthening these competencies would lead to improved job performance.

Future research should use longitudinal, multicentre or intervention‐based designs to determine whether changes in specific emotional intelligence competencies are associated with subsequent changes in nurses’ performance. Future studies should also incorporate supervisor‐rated or objective measures of performance and examine outcomes such as patient care quality, teamwork, workload, leadership and other organisational factors that may contribute to nurses’ job performance.

## 6. Relevance for Nursing Management Practice

The findings have implications for nursing management and continuing professional development. Self‐awareness and relationship management were independently associated with nurses’ self‐reported job performance in this study. Nursing managers may, therefore, consider incorporating activities that develop these competencies into continuing professional development programmes, particularly through reflective practice, interpersonal communication, conflict management and relationship‐building activities.

At the organisational level, emotional intelligence competencies may be incorporated into staff development, mentoring and leadership programmes. Such initiatives should be evaluated to determine whether strengthening these competencies is associated with changes in nurses’ performance and other relevant workplace outcomes. Given the cross‐sectional nature of the present study, recommendations for emotional intelligence development should be considered as practice implications rather than evidence that such interventions will cause improvements in job performance.

### 6.1. Limitations

This study has some limitations. The cross‐sectional design limits the interpretation of the findings to associations and does not allow causal conclusions about the relationship between emotional intelligence and nurses’ job performance. The study was also conducted at a single teaching hospital, so the findings may not represent nurses working in other healthcare settings.

Emotional intelligence and job performance were measured using self‐reported questionnaires. Responses may, therefore, have been affected by social desirability or common method bias, particularly because both the predictor and outcome measures were obtained from the same respondents. Although completed questionnaires were placed in envelopes before being submitted to unit in‐charges or shift leaders, this arrangement may still have influenced how some participants responded.

The study also measured nurses’ perceived job performance rather than performance assessed by supervisors or objective clinical indicators. Consequently, the findings do not show whether the observed associations extend to actual clinical performance, patient outcomes or teamwork effectiveness. Finally, the analysis did not capture other workplace factors that may be related to nurses’ performance, such as workload, staffing, leadership and burnout.

## Funding

The author did not receive support from any organisation.

## Disclosure

This article is based on the author’s master’s dissertation titled ‘Emotional Intelligence and Nurses’ Job Performance at The Cape Coast Teaching Hospital, Ghana’, submitted to the University of Cape Coast, Ghana. The dissertation has not been published in any peer‐reviewed journal. This manuscript represents a substantially revised and condensed version prepared for journal submission.

## Ethics Statement

Ethical approval was obtained from the Institutional Review Board of the Cape Coast Teaching Hospital before data collection commenced (Approval No. CCTHERC/EC/2021/084).

## Consent

Participation was voluntary, and written informed consent was obtained from all respondents. Participants were informed of their right to withdraw from the study at any stage without penalty.

## Conflicts of Interest

The author declares no conflicts of interest.

## Data Availability

The data that support the findings of this study are available from the corresponding author upon reasonable request.
